# Comparative Genomics of Acetic Acid Bacteria within the Genus *Bombella* in Light of Beehive Habitat Adaptation

**DOI:** 10.3390/microorganisms10051058

**Published:** 2022-05-20

**Authors:** Luca Härer, Maik Hilgarth, Matthias A. Ehrmann

**Affiliations:** 1 Chair of Microbiology, Technical University Munich, 85354 Freising, Germany; luca.haerer@tum.de; 2 Chair of Technical Microbiology, Technical University Munich, 85354 Freising, Germany; maik.hilgarth@tum.de

**Keywords:** *Bombella*, acetic acid bacteria, *Apis mellifera*, honeybee microbiota, comparative genomics, metabolism, adaption

## Abstract

It is known that the bacterial microbiota in beehives is essential for keeping bees healthy. Acetic acid bacteria of the genus *Bombella* colonize several niches in beehives and are associated with larvae protection against microbial pathogens. We have analyzed the genomes of 22 *Bombella* strains of different species isolated in eight different countries for taxonomic affiliation, central metabolism, prophages, bacteriocins and tetracycline resistance to further elucidate the symbiotic lifestyle and to identify typical traits of acetic acid bacteria. The genomes can be assigned to four different species. Three genomes show ANIb values and DDH values below species demarcation values to any validly described species, which identifies them as two potentially new species. All *Bombella* spp. lack genes in the Embden–Meyerhof–Parnas pathway and the tricarboxylic acid cycle, indicating a focus of intracellular carbohydrate metabolism on the pentose phosphate pathway or the Entner–Doudoroff pathway for which all genes were identified within the genomes. Five membrane-bound dehydrogenases were identified that catalyze oxidative fermentation reactions in the periplasm, yielding oxidative energy. Several complete prophages, but no bacteriocins, were identified. Resistance to tetracycline, used to prevent bacterial infections in beehives, was only found in *Bombella apis* MRM1^T^. *Bombella* strains exhibit increased osmotolerance in high glucose concentrations compared to *Gluconobacter oxydans*, indicating adaption to high sugar environments such as beehives.

## 1. Introduction

The majority of global fruit, vegetable or seed production of food crops depends on animal pollination, with the honeybee playing a key role [1,2]. Estimations suggest that a third of the food consumed comes from honeybee pollination [3]. Unfortunately, drastic reduction of honeybee populations were documented globally in recent years [4,5]. Versatile challenges such as pathogens, pesticides and anthropogenic factors are held responsible for this phenomenon [6,7]. This concerning development has drawn a focus on bee defense mechanisms, including symbiotic microbes with protective traits [8,9].

The core microbiome of the honeybee consists of a distinctive group of bacteria, which appears to have undergone long-term coevolution [10]. A member of this group is the acetic acid bacteria *Bombella* (previously also invalidly designated to as *Parasaccharibacter* or “Alpha 2.2”), a genus composed of four described species, namely *B. apis*, *B*. *intestini*, *B. favorum* and *B. mellum* [11,12,13,14]. Focus has been drawn to this genus, since it was shown to have a positive impact against fungal pathogens [15,16]. Bacteria of that genus colonize not only the intestines of the worker bees, such as the crop and hindgut, but different niches in the beehive, such as the larvae or the gut of the queen [17,18].

The genomes of *Bombella* spp. harbor several features associated with the transition to honeybee symbiosis, but they also exhibit typical traits of acetic acid bacteria, such as membrane-bound dehydrogenases for oxidative fermentation [19,20]. By comparative genomic analysis of available *Bombella* genomes, this study aims to further elucidate central metabolism of this genus but also highlights features that are likely a result of co-evolution in the honeybee environment. Findings of this study will help to further understand the beehive ecosystem, which has a decisive influence on global cultivation of food crops.

## 2. Materials and Methods

### 2.1. Isolation and Ecology

Strains were isolated from *Apis mellifera* honeycombs in Freising (Germany) and from *Apis mellifera* honeycombs in Salzburg (Austria). Isolation was performed as described in a previous study by Hilgarth et al. [13].

### 2.2. Genome Sequencing, Assembly and Annotation

Genomes of *Bombella apis* TMW 2.1882, TMW 2.1884, TMW 2.1886, TMW 2.1888, TMW2.1890 and TMW2.1891 were sequenced via an Illumina MiSeq platform. SPAdes version 3.90 was used for the construction of assemblies [21]. CheckM [22] and ContEst16S [23] were used to assess the quality of the assemblies. The NCBI PGAP pipeline was used for genome annotations [24].

### 2.3. 16S rRNA Gene Sequence, Average Nucleotide Identity (ANI) and In Silico DNA-DNA Hybridization (DDH) Values

Sequences of complete 16S rRNA genes were obtained from whole genome sequences. Sequence analysis and calculation of phylogenetic trees was performed with MEGA7 [25], and the integrated ClustalW was used for sequence alignment. ANIb and ANIm algorithms implemented in the JspeciesWS web service were used to calculate the average nucleotide identity (ANI) [26,27]. In silico DDH was calculated using the Genome-to-Genome Distance Calculator’s (GGDC) subspecies concept [28] as described in Werum et al. [29]. Species delineation cutoffs for ANI values are 95–96% [30] and 70% for DDH values [31,32].

### 2.4. Comparative Genomic Analysis

To identify differences in available *Bombella* spp. genomes, the BlAst Diagnostic Gene finder (BADGE) tool was used with default settings and a sequence identity cut-off of 95% [33]. The output was used for core and pan genome calculations and was visualized with the BLAST ring image generator (BRIG) [34]. Annotations of interest were manually checked with RAST annotations [35] or the InterPro web tool [36].

Genomes were screened for prophage sequences using the PHASTER web tool (PHAge Search Tool Enhanced Release) [37]. The BAGEL4 web server was used to identify potential bacteriocins in the *Bombella* genomes [38]. Secondary metabolite biosynthesis gene clusters were identified with the antiSMASH webtool [39].

### 2.5. Analysis of Antimicrobial Resistance

The resistance of available *Bombella* spp. was tested against twelve antibiotics (Table S4). A disk diffusion method was adapted from the “European Committee on Antimicrobial Susceptibility” testing (EUCAST) guidelines. Overnight cultures were spread onto LMG404 media agar plates (50 g L^−1^ glucose; 10 g L^−1^ yeast extract, pH 6.5), and antibiotic discs from Oxoid Ltd. (UK) were placed onto the plates. The diameter of the growth inhibition zone was measured after 48 h of incubation at 30 °C at oxic conditions.

### 2.6. Growth Experiments

The growth of available *Bombella* spp. on glucose, D-fructose, sucrose and D-mannitol was monitored on agar plates containing 50 g L^−1^ of the respective carbohydrate, 10 g L^−1^ yeast extract and 15 g L^−1^ agar–agar. Plates were incubated at 30 °C over 48 h. Plates without added carbohydrate were used as a negative control.

The maximal growth rate (*µ*_max_) of available strains was measured at 100 and 300 g L^−1^ glucose. The medium used consisted only of 10 g L^−1^ yeast extract in addition to glucose. Overnight cultures were washed in saline (0.9% NaCl) and used for inoculation (5%). The cultures were split into three 250 µL cultivations in 96-well plates. Cultivation was performed in a plate photometer at 30 °C, and the cells were shaken at 500 rpm. The optical density was monitored at 600 nm over 60 h. R package “grofit” was used to calculate *µ*_max_ [40].

## 3. Results

### 3.1. Phylogeny, Current Taxonomy and Reclassification of Parasaccharibacter Apium, Saccharibacter *sp.* and Bombella *sp.* Strains

**Figure 1:**
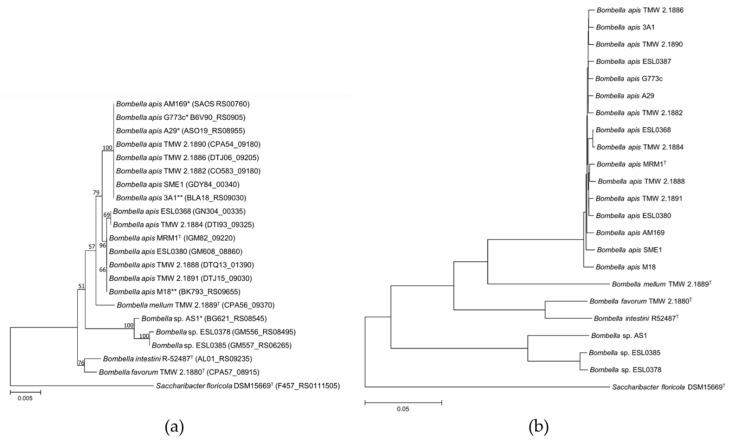
(**a**) Neighbor-joining (NJ) tree based on partial 16S rRNA gene sequences and (**b**) phylogenetic tree based on ANI-values via ANIb algorithm as implemented in JSpeciesWS. *Saccharibacter floricola* was used as an outgroup. Locus tags of partial 16S rRNA gene sequences are shown in parentheses. * previously designated as “*Parasaccharibacter*”; ** previously designated as “*Saccharibacter*”.

Genomes of our isolates and available strains within the NCBI database (Table 1) were subjected to phylogenetic analyses based on partial 16S rRNA genes (Figure 1A), ANIb values (Figure 1B), and in silico DDH distances (Table S1). Strains “*Parasaccharibacter apium*” B8 and C6 were removed from the analysis due to 100% ANIb identity to A29 indicating clonal origin. Strains ESL0378 (Accession number: GM556_RS08495) and ESL0385 (Accession number: GM557_RS06265) exhibit ANIb and in silico DDH values of 99.1% and 91.8% to each other, but 73.6–74.4% and 18.6–19.1% to other known type strains of *Bombella*. These values are below the species demarcation threshold and are identified as two strains of a novel and hitherto undescribed species of the genus *Bombella*.

### 3.2. Origin and General Genomic Features of Bombella *sp.* Isolates

Isolation origin as well as general genome statistics are listed in Table 1. *Bombella* sp. strains have been hitherto isolated from *Apis mellifera*, with the exception of *Bombella intestini*, which was isolated from *Bombus lapidaries* [12]. In this study, we report the first isolation of *Bombella apis* (TMW 2.1886 and TMW 2.1888) from *Apis mellifera mellifera*, and its royal jelly. *Bombella* strains have been isolated from different parts of the beehive (bee gut, stomach, crop, larva, honey) as well as from different geographic locations (Germany, Switzerland, Austria, Italy, Hungary, Belgium, United States of America, South Korea). However, all of these strains form a homogenous group of *Bombella apis*, absent of subgroups within the species as exhibited by similar differences in their ANIb values (Figure 1), indicating their global affiliation to the autochthonous beehive microbiota. Furthermore, we have isolated different species (*B. apis*, *B. favorum* and *B. mellum*) from the same beehive, indicating the high diversity as well as a general adaptation to symbiosis with bees.

**Figure 2:**
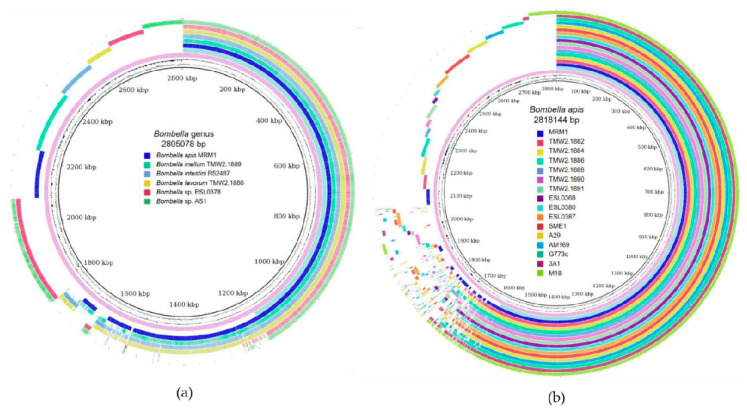
BRIG output image based on the nucleotide sequences of (**a**) *Bombella* species and (**b**) *Bombella apis* strains. (█) pan genome; (█) core genome.

The genomic G+C content (%) and genome size (mb) is visualized in Figure S1. While genome size of *Bombella* isolates are quite similar (1.85–2.01), the G+C content showed a high degree of variance between the species, from 52.64% (*Bombella* sp. AS1) to 60.43% (*Bombella mellum*). No plasmids could be identified within the genomes. A visualization of the core, accessory and pangenome is shown in Figure 2 of each type of strain of the *Bombella* genus (Figure 2A) and all *Bombella apis* strains (Figure 2B). The core genome of the genus (approx. 1.15 mbp) comprises approx. half of the respective-type strain genome. Within the accessory genome, different subgroups are formed: *Bombella* sp. ESL0378 *Bombella* sp. AS1, *Bombella favorum*/*Bombella intestini*; *Bombella mellum*/*Bombella apis*, which also resembles the phylogenetic relationship between the species.

The core genome of *Bombella apis* strains comprises approx. 80% (approx. 1.6 of 2 mb) of the average *Bombella apis* genome. No apparent subgroups were visible within the accessory genomes supporting the homogenous clustering within the ANIb analysis.

### 3.3. Main Cytoplasmic Carbohydrate Metabolism

**Figure 3:**
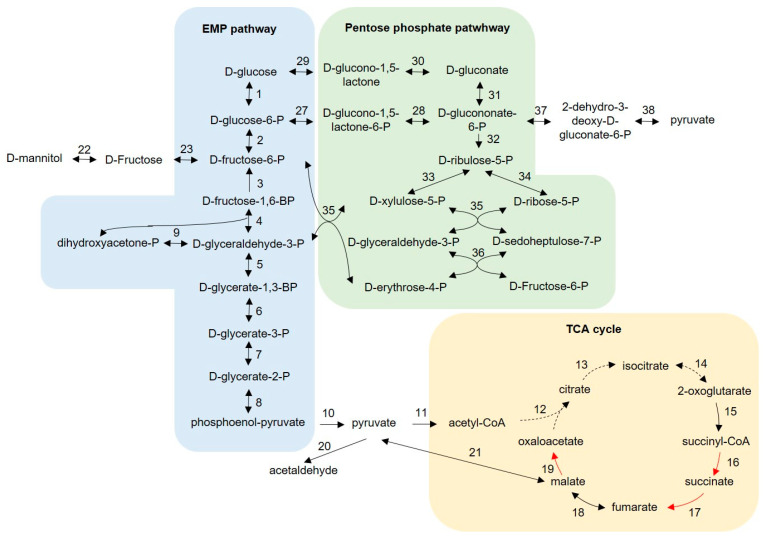
Predicted cytoplasmic carbohydrate metabolism of *Bombella* spp. (based on [19,45]). Dashed arrows indicate genes not present in all genomes. Red arrows indicate missing genes. Locus tags are summarized in Table S2. 1: glucokinase; 2: glucose-6-phosphate isomerase; 3: fructose-1,6-bisphosphatase; 4: fructose-bisphosphate aldolase; 5: glyceraldehyde-3-phosphate dehydrogenase; 6: phosphoglycerate kinase; 7: phosphoglycerate mutase; 8: enolase; 9: triose-phosphate isomerase; 10: pyruvate kinase; 11: pyruvate dehydrogenase complex; 12: citrate synthase; 13: aconitate hydratase; 14: isocitrate dehydrogenase; 15: oxoglutarate dehydrogenase complex; 16: succinyl-CoA synthetase; 17: succinate dehydrogenase; 18: fumarase; 19: malate dehydrogenase; 20: pyruvate decarboxylase; 21: malate dehydrogenase (oxaloacetate-decarboxylating); 22: mannitol 2-dehydrogenase; 23: fructokinase; 24: glucose-6-phosphate dehydrogenase; 25: 6-phosphogluconolactonase; 26: glucose 1-dehydrogenase; 27: gluconolactonase; 28: gluconoate kinase; 29: phosphogluconate dehydrogenase; 30: ribulose-phosphate 3-epimerase; 31: ribose-5-phosphate isomerase; 32: transketolase; 33: transaldolase; 34: 6-phosphogluconate dehydratase; 35: 2-dehydro-3-deoxyphosphogluconate aldolase.

The Embden–Meyerhof–Parnas (EMP) pathway, the tricarboxylic acid (TCA) cycle and the pentose phosphate pathway were reconstructed to elucidate cytoplasmic carbohydrate catabolism in *Bombella* spp. (Figure 3, Table S2). All genomes lack a phospho-fructokinase gene, making the EMP pathway incomplete. The presence of a fructose-1,6-bisphosphatase gene (Enzyme 3, Figure 3) should enable *Bombella* spp. to perform gluconeogenesis. All genes encoding the enzymes of the pentose phosphate way were identified. Genes encoding for 6-phosphogluconate dehydratase and 2-dehydro-3-deoxyphosphogluconate aldolase complete the Entner–Doudoroff (ED) pathway. No genes encoding for succinyl-CoA synthetase, succinate dehydrogenase or malate dehydrogenase were identified, suggesting an incomplete TCA cycle. *Bombella favorum*, *Bombella* sp. ESL0378, *Bombella* sp. ESL0385 and *Bombella* sp. AS1 additionally lack genes for the enzymes citrate synthase, aconitate hydratase and isocitrate dehydrogenase. Genes for the incorporation of D-fructose and D-mannitol into the intracellular carbohydrate metabolism were identified and confirmed in growth experiments. In addition, a gene was identified that encodes for an extracellular enzyme with invertase activity (GH32 family) and should enable growth of *Bombella* spp. on sucrose. To confirm the in silico findings, growth of available *Bombella* spp. was verified on agar plates with 50 g L^−1^ D-fructose, D-mannitol or sucrose as the main carbon source. For all combinations of strains and carbohydrates, growth could be observed after 48 h.

### 3.4. Membrane-Bound Dehydrogenases and Respiratory Enzymes

Four membrane-bound dehydrogenases (DH) were identified in all analyzed *Bombella* genomes (Table S2). A PQQ-dependent glucose DH and a gluconate-2 DH catalyze the oxidation from glucose to gluconate to 2-keto-gluconate. Additionally, all strains harbored a quinone-dependent dihydroorotate DH and a D-lactate DH. A fifth membrane-bound alcohol DH gene with an undefined substrate spectrum was identified in duplicates for all genomes except for *Bombella* sp. A1, *Bombella* sp. ESL0378 and *Bombella* sp. ESL0385, where single, truncated versions were identified. One of the two copies in *B. apis* G773c appears not to be functional due to a frame shift. The respiratory chain of *Bombella* spp. consists of an NADH DH (type II), a bo3-type cytochrome c oxidase, a cytochrome bc1 complex, a flavoprotein-ubiquinone oxidoreductase and a cytochrome D ubiquinol oxidase. The only exception is *B*. *intestini*, where no cytochrome D ubiquinol oxidase was identified.

### 3.5. Presence of Prophages and Bacteriocins

Prophages in the *Bombella* genomes were identified using the PHASTER web tool. Complete phages were identified for *B. apis* ESL0387, *B. apis* SME1, *B. apis* TMW 2.1882 and *B. apis* G773c. In the genomes of *B. apis* MRM1^T^, *B. apis* ESL0387, *B. apis* SME1, *B. apis* TMW 2.1884 and *B. favorum* TMW 2.1880 prophages were identified that were labeled as questionable. The BAGEL4 web server was used to identify harbored bacteriocins. No bacteriocins were found.

### 3.6. Polyketide Synthase Gene Cluster

A type 1 polyketide synthase gene cluster (T1PKS) was recently associated with potential antifungal properties in *B. apis* [16]. The gene cluster was identified in all *Bombella* genomes, but also in other acetic acid bacteria such as *Saccharibacter floricola* DSM 15669^T^, *Gluconacetobacter diazotrophicus* PA1 5 and *Asaia bogorensis* NBRC 16594 (Table S3).

### 3.7. Tetracycline Resistance

The *Bombella apis* MRM1^T^ genome carries two genes associated with resistance to tetracycline antibiotics: a tetracycline resistance transcriptional repressor (*tetR*; IGM82_03615) and a tetracycline efflux MFS transporter (*tetG*; IGM82_03620). No other genes associated with tetracycline resistance were identified. In order to confirm the resistance, available strains were tested for resistance toward tetracycline (30 µg) and doxycycline (30 µg) via a disk diffusion method. Growth inhibition zones varied between 27 and 36 mm for tetracycline and from 23 to 31 mm for doxycycline. No growth inhibition was measured for *Bombella apis* MRM1^T^, confirming the in silico finding of the resistance genes *tetR* and *tetG*.

### 3.8. Adaptation to High Glucose Concentration

**Figure 4:**
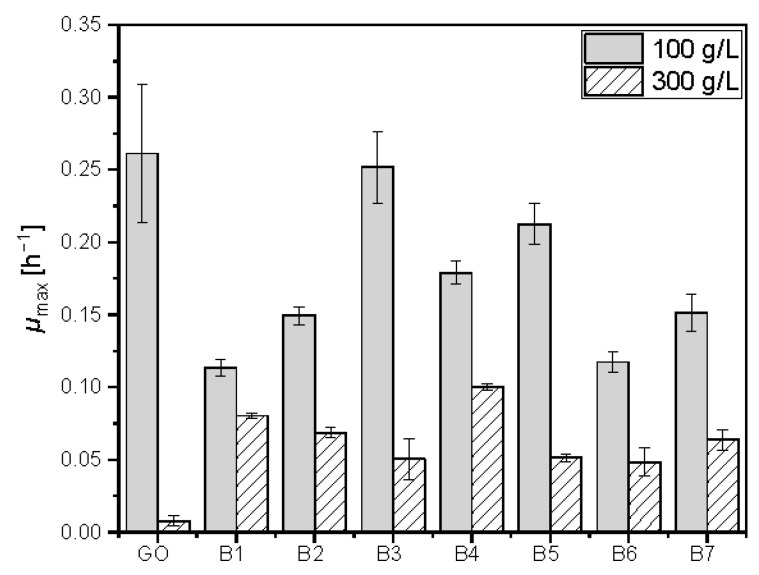
Maximal growth rates (*µ*_max_) of *Bombella* strains and *Gluconobacter oxydans* DSM46615 (GO) at 100 and 300 g L^−1^ glucose at 30 °C. B1: *B. favorum* TMW 2.1880; B2: *B. apis* TMW 2.1882; B3: *B. apis* TMW 2.1884; B4: *B. apis* TMW 2.1886; B5: *B. apis* TMW 2.1888; B6: *B. apis* TMW 2.1890; B7: *B. apis* TMW 2.1891.

It can be assumed that the symbiotic lifestyle of *Bombella* sp. in the honeybee environment led to an evolutionary adaption toward high sugar concentrations. Growth experiments at 100 and 300 g L^−1^ glucose were performed with available *Bombella* strains and were compared to *Gluconobacter oxydans* DSM46615, a related acetic acid bacterium. The maximal growth (*µ*_max_) rates are plotted in Figure 4. While *µ*_max_ of *Gluconobacter oxydans* DSM46615 at 100 g L^−1^ glucose is among the highest, at 300 g L^−1^, it is multiple times lower than *µ*_max_ of the *Bombella* strains. The higher tolerance toward osmotic pressure supports the thesis of a strong adaptation of *Bombella* strains toward sugar-rich environments, such as honey beehives.

## 4. Discussion

### 4.1. Phylogeny, Current Taxonomy and Reclassification of Parasaccharibacter apium, Saccharibacter *sp.* and Bombella *sp.* Strains

The taxonomy of *Bombella* strains has previously been inconsistent due to the affiliation to the non-valid “*Parasaccharibacter*” or to “*Saccharibacter* sp.”. Recently, strains previously designated as “*Parasaccharibacter apium*” (strains A29, B8, C6, G773c) and “*Saccharibacter* sp.” (Strains 3A1, M18, AM169) were proposed to be reclassified into *Bombella apis* based on species demarcation values. Furthermore, it was proposed to not use non-valid taxon “*Parasaccharibacter*” [46]. We can confirm that the ANIb values of the proposed *B. apis* strains (including strains previously designated *B. apis*) and the type strain MRM1^T^ are 98.7–99.3%. The values are clearly above the species delineation cutoff of <95–96% (16S rRNA similarity >99.7%), indicating unified affiliation to one species. Additionally, in silico DDH values of proposed *B. apis* strains to the type strain are 89.6–94.6% and are therefore clearly above the species cutoff of <70%. Additionally, we can confirm that strain AS1 (published within the NCBI database as *Parasaccharibacter apium* AS1) exhibits ANIb and in silico DDH values of 73.6–74.4% and 18.5–18.9% to known type strains of *Bombella*, respectively, and therefore represents a novel species of the genus *Bombella* and should be reclassified to *Bombella* sp. AS1. Compared to strains ESL0378 and ESL0385, the ANIb and in silico DDH values of *Bombella* sp. AS1 are 92.1/92.2% and 47.3/47.4%. From this, it can be concluded that these strains belong to separate hitherto undescribed species of the genus *Bombella*.

### 4.2. Predictive Carbohydrate Metabolism of Bombella *spp.*

The cytoplasmic carbohydrate metabolism of *Bombella* spp. was predicted by comparative genomic analysis (Figure 3), including the Embden–Meyerhof–Parnas (EMP) pathway, the pentose phosphate pathway (PPP) and the tricarboxylic acid (TCA) cycle. All genomes lack genes for phospho-fructokinases, disrupting the EMP pathway. Incomplete glycolysis was already described for several acetic acid bacteria such as *Gluconobacter oxydans* and *Acetobacter pasteurianus* [45,47,48].

We assume that intracellular glucose is mainly metabolized via the PPP or the Entner–Doudoroff (ED) pathway for which all genes were identified. This cytoplasmic carbon flux was verified for related *G. oxydans* 621H by a ^13^C-based metabolic flux analysis [49]. Intracellular D-mannitol and D-fructose can also be incorporated in this flux via a mannitol dehydrogenase and a fructokinase.

*Bombella* genomes lack three or, in the case of *Bombella* sp. ESL0378, *Bombella* sp. ESL0385 and *Bombella* sp. AS1, six genes of the TCA cycle. An incomplete TCA cycle missing three genes is described for other acetic acid bacteria [45,50,51]. In that case, all steps until the synthesis of succinyl-CoA are present. To prevent an accumulation of TCA-cycle intermediates, it is assumed that it is regulated to only meet the cellular demand of 2-oxoglutarate as a precursor for biosynthesis of the glutamate family of amino acids [49]. Other intermediates of the TCA cycle can derive from different sources, e.g., amino acids. For *Bombella intestine*, theoretical pathways linked to L-asparagine were constructed that would result in succinate, fumarate or oxaloacetate [19]. No studies were found that deal with acetic acid bacteria missing six genes of the TCA cycle, and hence, further investigations would be necessary to elucidate cellular mechanisms.

Acetic acid bacteria are well known for their ability to incompletely oxidize sugars and alcohols in the periplasm in so-called oxidative fermentations [52]. The responsible membrane-bound dehydrogenases (DH) are coenzyme-dependent and are coupled to the respiratory chain [52,53]. For *G. oxydans*, it was shown that most of the glucose in the medium is not taken up by the cell but is oxidized in the periplasm [49]. Five membrane-bound DHs were identified in *Bombella* spp., namely a PQQ-dependent glucose DH, a gluconate-2 DH, a quinone-dependent dihydroorotate DH, a D-lactate DH and an alcohol DH gene with an undefined substrate spectrum. Prediction of the substrate spectrum is not expedient, since it was shown that broad spectra of substrates are accepted in comparable enzymes [54]. Previously, a related enzyme was annotated as a glycerol, sorbitol and glycerol DH [45]. Other identified electron donors of the respiratory chain were a type II NADH DH and a flavoprotein–ubiquinone oxidoreductase with unknown substrate spectrum. In contrast, *Gluconobater oxydans* possesses 32 membrane-bound DH [45,55]. The reduced amount of membrane-bound DH in *Bombella* spp. and the focus on glucose/gluconate oxidation might be an indicator of the adaption to the honeybee environment, where glucose is constantly available [56,57].

### 4.3. Potential Role of Extracellular Invertase in Melezitose Degradation

The “honeydew flow disease” in honeybees results from the feed on honeydew and impacts whole colonies. It is linked to the presence of the honeydew trisaccharide melezitose [58]. Melezitose is hydrolyzed to some extent in the bee gut by enzymes with invertase activity, expressed either by the microbiome organisms or by the bee itself, but accumulation of the sugar could lead to severe symptoms [58]. It was shown that *Bombella apis* was one of the microbiome bacteria that was not negatively influenced by a melezitose feed [58]. This phenomenon might be linked to the expression of an extracellular invertase present in all *Bombella* spp. analyzed, which carries a twin-arginine translocation (TAT) signal for secretion and a glycoside hydrolase family 32 (GH32) motif. The catalytic activity of such enzymes in the bee environment might be crucial to prevent melezitose accumulation and the “honeydew flow disease” associated with it.

### 4.4. Potential Antifungal Properties

*Bombella apis* is associated with the protection of honey beehives from fungal pathogens [16]. It is presumed that an antifungal metabolite is responsible for the protective effect, probably synthesized by enzymes of a type 1 polyketide synthase gene cluster [16]. All *Bombella* genomes contain such a cluster, but it was also identified in other acetic acid bacteria such as *Saccharibacter floricola* DSM 15669, *Gluconacetobacter diazotrophicus* PA1 5 and *Asaia bogorensis* NBRC 16594 (Table S3). The wide distribution of the type 1 polyketide synthase gene cluster in other acetic acid bacteria indicates that it is not a genetic feature that is associated with the symbiosis of *Bombella* spp. and honeybees.

The antifungal properties of *Bombella apis* might not be caused by the synthesis of a secondary metabolite, but it could also be a result of primary metabolism. For example, it would be possible that the extracellular accumulation of gluconate via oxidative fermentation or secretion of acetate could have an impact on fungal growth. Separate alterations of the surroundings could accumulate to the measured decrease in fungal growth.

### 4.5. Tetracycline Resistance

The resistance of *Bombella apis* MRM1^T^ toward tetracycline and doxycycline was shown in a disk diffusion assay (Table S4). Tetracycline is a broad-spectrum antibiotic used in non-EU countries to prevent microbial infections with, e.g., *Paenibacillus larvae,* a bacteria responsible for foulbrood disease [59]. It was shown that tetracycline has negative effects on the size and composition of the honeybee gut microbiome and on the resistance of honeybees toward other opportunistic bacterial pathogens [60].

The genomes of *Bombella* spp. were analyzed for genes associated with tetracycline resistance. No genes were found, except for a tetracycline resistance transcriptional repressor (*tetR*; IGM82_03615) and a tetracycline efflux MFS transporter (*tetG*; IGM82_03620) in *Bombella apis* MRM1^T^. Both genes are located in proximity, and the transcriptional repressor TetR likely regulates the expression of the efflux-MFS transporter dependent on the presence of a tetracycline antibiotic [61]. The most common mechanism of tetracycline resistance is active efflux of the drug and numerous genetic determinants have been described. The *tetG* gene we identified in *Bombella* is most clearly related (95% similarity) to the tetG of the Gram-negative sewage isolate *Paradevosia shaoguanensis* J5–3T, which has also been described as tetracycline resistant [62].

When using antibiotics in beekeeping, the spread of resistance genes could be promoted. The lacking resistance of the majority of *Bombella* spp. and of other organisms of the honeybee microbiome to antibiotics should be considered when using such agents for prevention purposes.

### 4.6. Sugar Tolerance

*Bombella* spp. are described to grow in media containing 50% of glucose [13], a trait likely linked to the sugar-rich habitat as a honeybee symbiont. We compared the maximal growth rate of available *Bombella* strains with *G. oxydans* DSM46615 at 100 and 300 g L^−1^ in order to illustrate the high osmotolerance of *Bombella* spp. A main mechanism for prokaryotes to balance osmotic pressure is the accumulation of compatible solutes in the cytoplasm [63]. In *G. oxydans*, the accumulation of mannitol is a key mechanism of osmoprotection and is linked to the presence of mannitol dehydrogenases [64]. This mechanism might also be relevant in *Bombella* spp. since a mannitol dehydrogenase gene was identified in all analyzed genomes (Table S2). As shown for *Gluconacetobacter diazotrophicus*, osmoprotection in acetic acid bacteria is a multifactorial interplay of different cellular mechanisms [65]. Osmotic stress influences nutrient uptake, de novo saturated fatty acids biosynthesis, cell division and other factors, an impact too complex to predict by comparative genomic analysis alone, which should be the subject of future physiologic studies.

## 5. Conclusions

The comparative genome analysis of 22 *Bombella* spp. genomes revealed typical features of acetic acid bacteria with strong adaptions to the symbiotic lifestyle in the beehive habitat. The carbohydrate metabolism of *Bombella* spp. appears to be adapted to the constant availability of glucose, fructose and sucrose in high concentrations. Cellular mechanisms from which the host might benefit have been discussed and provide a basis for future research and a better understanding regarding the interaction of the honeybee and its symbiotic microbiome.

## Figures and Tables

**Table 1:** Characteristics, origin and isolation source of available *Bombella* genomes within the NCBI database.

Designation	Contigs	Size (mbp)	GC (%)	Density (%)	Source	Symbiotic Host	Country	Reference	BioSample
*Bombella apis* MRM1^T^	7	2.03	59.59	90.55	midgut	*Apis mellifera*	KOR	[11]	SAMN16262074
*Bombella apis* ESL0368	1	1.99	59.6	89.85	gut of adult queen	*Apis mellifera*	CHE	[41]	SAMN13280441
*Bombella apis* ESL0380	10	1.98	59.57	90.43	gut of adult queen	*Apis mellifera*	CHE	[41]	SAMN13280444
*Bombella apis* ESL0387	10	2.04	59.47	90.3	gut of adult queen	*Apis mellifera*	CHE	[41]	SAMN13280447
*Bombella apis* SME1	11	2.09	59.56	90.09	beehive	*Apis mellifera*	USA	-	SAMN13042715
*Bombella apis* TMW 2.1882	5	2.02	59.41	90.33	honey	*Apis mellifera*	DEU	this study	SAMN07674798
*Bombella apis* TMW 2.1884	19	2.05	59.37	90.33	honey	*Apis mellifera*	DEU	this study	SAMN09635582
*Bombella apis* TMW 2.1886	39	2.05	58.86	89.57	honey	*Apis mellifera mellifera*	AUT	this study	SAMN09635591
*Bombella apis* TMW 2.1888	1	2.01	59.48	89.72	royal jelly	*Apis mellifera mellifera*	AUT	this study	SAMN09641705
*Bombella apis* TMW 2.1890	7	2.02	59.49	90.29	honey	*Apis mellifera*	DEU	this study	SAMN07675059
*Bombella apis* TMW 2.1891	7	2.01	59.44	90.46	honey	*Apis mellifera*	DEU	this study	SAMN09635595
*Bombella apis* A29	27	2.01	59.39	90.15	larva	*Apis mellifera*	USA	[14]	SAMN04240487
*Bombella apis* AM169	9	1.98	59.32	90	gut adult	*Apis mellifera*	ITA	[42]	SAMEA3139036
*Bombella apis* G773c	1	2.01	59.42	90.02	hindgut adult	*Apis mellifera*	USA	[43]	SAMN06649799
*Bombella apis* 3A1	24	2.01	59.41	90.01	honey	*Apis mellifera*	HUN	[44]	SAMN05935507
*Bombella apis* M18	11	2.08	59.35	90.22	stomach adult	*Apis mellifera*	HUN	[44]	SAMN05935506
*Bombella intestini* R52487^T^	12	2.02	54.94	90.16	crop	*Bombus lapidarius*	BEL	[19]	SAMN02598725
*Bombella favorum* TMW 2.1880^T^	7	1.98	55.33	90.29	honey	*Apis mellifera*	DEU	[13]	SAMN07674723
*Bombella mellum* TMW 2.1889^T^	11	2.07	60.43	89.93	honey	*Apis mellifera*	DEU	[13]	SAMN07674951
*Bombella* sp. ESL0378	15	1.85	52.88	91.34	gut of adult worker	*Apis mellifera*	CHE	[41]	SAMN13280442
*Bombella* sp. ESL0385	5	1.9	52.91	91.51	gut of adult worker	*Apis mellifera*	CHE	[41]	SAMN13280446
*Bombella* sp. AS1	13	1.85	52.64	91.55	larva	*Apis mellifera*	USA	-	SAMN05720096

## Data Availability

Not applicable.

## Supplementary Materials

The following supporting information can be downloaded at: https://www.mdpi.com/article/10.3390/microorganisms10051058/s1, Figure S1: Visualization of the genomic G+C content (%) over the genome size (mb) of *Bombella* genomes. Black dot: *Bombella* sp. AS1; Grey dots: *Bombella* sp. ESL0378 / ESL 0385; Red dot: *Bombella favorum*; Green dot: *Bombella intestini*; Blue dots: *Bombella apis*; Purple dot: *Bombella mellum*; Table S1: In silico DDH distances of analyzed *Bombella* genomes; Table S2: Locus tags of predicted central carbohydrate metabolism and membrane-bound dehydrogenases in *Bombella* spp.; Table S3: Genome accessions for contigs of type 1 polyketide synthase gene clusters; Table S4: Growth inhibition zone diameter (in mm) of antibiotic disc diffusion assay.
