# Supplementary material for: Comparative Genomics of Acetic Acid Bacteria within the Genus Bombella in Light of Beehive Habitat Adaptation
Source: Microorganisms. 2022 May 20;10(5):1058. doi: 10.3390/microorganisms10051058 (PMC9147383; doi:10.3390/microorganisms10051058)
Supplement: Supplementary file 1 [file microorganisms-10-01058-s001.zip › Figure S1.pdf]

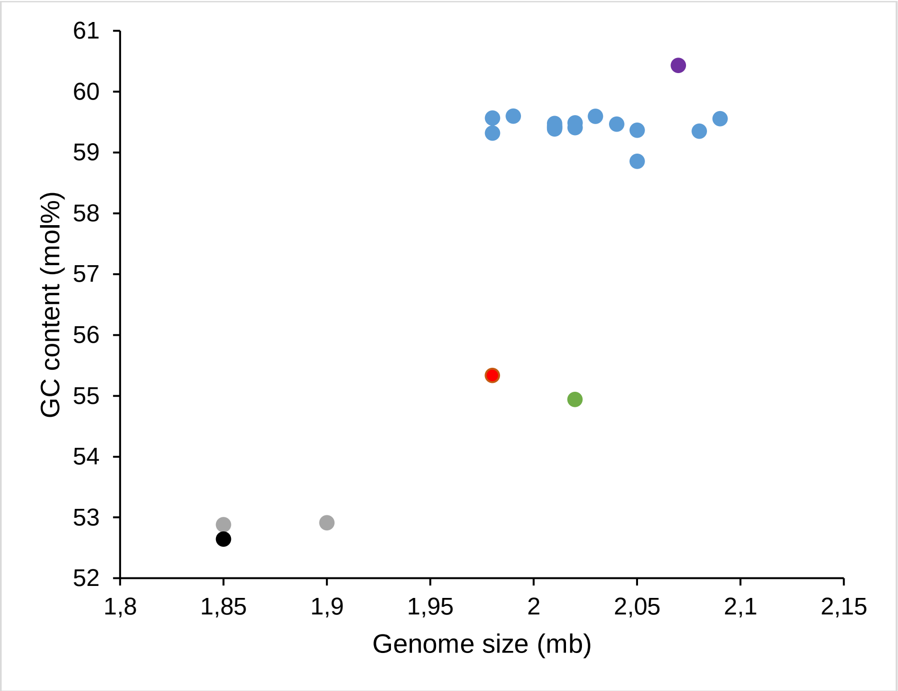

Figure S1: Visualization of the genomic G+C content (mol%) over the genome size (mb) of *Bombella* genomes. Black dot: *Bombella* sp. AS1; Grey dots: *Bombella* sp. ESL0378 / ESL 0385; Red dot: *Bombella favorum*; Green dot: *Bombella* intestini; Blue dots: *Bombella apis*; Purple dot: *Bombella mellum*.
