# Supplementary material for: Comparative Genomics of Acetic Acid Bacteria within the Genus Bombella in Light of Beehive Habitat Adaptation
Source: Microorganisms. 2022 May 20;10(5):1058. doi: 10.3390/microorganisms10051058 (PMC9147383; doi:10.3390/microorganisms10051058)
Supplement: Supplementary file 1 [file microorganisms-10-01058-s001.zip › Table S1.pdf]

Table S1: *In silico* DDH distances of analyzed *Bombella* genomes

		Formula 1				Formula 2				Formula 3				G+C difference
Query genome	Reference genome	DDH	Model C.I.	Distance	Prob. DDH >= 70%	DDH	Model C.I.	Distance	Prob. DDH >= 70%	DDH	Model C.I.	Distance	Prob. DDH >= 70%	
Bombella_apis_30666T	Bombella_apis_ESL0368	97.2	[95.6 - 98.2%]	0.0357	99.09	94	[92.3 - 95.4%]	0.0077	96.97	98.1	[97 - 98.8%]	0.0431	99.94	0.01
Bombella_apis_30666T	Bombella_apis_ESL0380	96.3	[94.4 - 97.6%]	0.0423	98.93	94.6	[92.9 - 95.9%]	0.0071	97.1	97.6	[96.3 - 98.4%]	0.0491	99.93	0.03
Bombella_apis_30666T	Bombella_apis_ESL0387	92.6	[89.8 - 94.7%]	0.0661	98.1	93.9	[92.1 - 95.3%]	0.0079	96.93	95	[93 - 96.4%]	0.0734	99.83	0.13
Bombella_apis_30666T	Bombella_apis_SME1	92.8	[90 - 94.9%]	0.0649	98.15	93.4	[91.5 - 94.9%]	0.0084	96.8	95	[93.1 - 96.5%]	0.0728	99.84	0.04
Bombella_apis_30666T	Bombella_apis_TMW21882	93.7	[91.1 - 95.6%]	0.0595	98.38	94.2	[92.4 - 95.5%]	0.0075	97.01	95.8	[94 - 97%]	0.0666	99.87	0.19
Bombella_apis_30666T	Bombella_apis_TMW21884	94.7	[92.3 - 96.4%]	0.0534	98.6	93.8	[91.9 - 95.2%]	0.008	96.9	96.4	[94.8 - 97.5%]	0.061	99.89	0.22
Bombella_apis_30666T	Bombella_apis_TMW21886	94.1	[91.5 - 95.9%]	0.0573	98.46	94.2	[92.4 - 95.5%]	0.0076	97	96	[94.3 - 97.2%]	0.0644	99.88	0.73
Bombella_apis_30666T	Bombella_apis_TMW21888	97.8	[96.4 - 98.6%]	0.031	99.19	94.2	[92.5 - 95.6%]	0.0075	97.02	98.5	[97.5 - 99%]	0.0382	99.95	0.12
Bombella_apis_30666T	Bombella_apis_TMW21890	94.8	[92.5 - 96.5%]	0.0525	98.63	93.8	[91.9 - 95.2%]	0.008	96.9	96.5	[94.9 - 97.6%]	0.0601	99.9	0.1
Bombella_apis_30666T	Bombella_apis_TMW21891	96.8	[95 - 97.9%]	0.0388	99.02	94.1	[92.4 - 95.5%]	0.0076	96.99	97.8	[96.7 - 98.6%]	0.0461	99.94	0.15
Bombella_apis_30666T	Parasaccharibacter_apium_A29	93.7	[91.1 - 95.6%]	0.0593	98.38	94.3	[92.5 - 95.6%]	0.0074	97.03	95.8	[94.1 - 97%]	0.0663	99.87	0.21
Bombella_apis_30666T	Parasaccharibacter_apium_AM169	95.3	[93.1 - 96.9%]	0.0492	98.73	93.7	[91.8 - 95.1%]	0.0081	96.88	96.8	[95.3 - 97.8%]	0.057	99.91	0.27
Bombella_apis_30666T	Parasaccharibacter_apium_G773c	94	[91.5 - 95.9%]	0.0575	98.45	94.6	[93 - 95.9%]	0.007	97.12	96	[94.4 - 97.2%]	0.0641	99.88	0.18
Bombella_apis_30666T	Saccharibacter_sp_3A1	95.4	[93.2 - 96.9%]	0.0486	98.75	93.7	[91.9 - 95.2%]	0.0081	96.89	96.9	[95.4 - 97.9%]	0.0563	99.91	0.18
Bombella_apis_30666T	Saccharibacter_sp_M18	94	[91.5 - 95.8%]	0.0577	98.45	89.6	[87.2 - 91.5%]	0.0126	95.65	95.4	[93.6 - 96.8%]	0.0696	99.86	0.24
		Formula 1				Formula 2				Formula 3				G+C difference
Query genome	Reference genome	DDH	Model C.I.	Distance	Prob. DDH >= 70%	DDH	Model C.I.	Distance	Prob. DDH >= 70%	DDH	Model C.I.	Distance	Prob. DDH >= 70%	
Bombella_sp_ESL0385	Bombella_apis_30666T	20.7	[17.5 - 24.3%]	0.6926	0	18.7	[16.5 - 21%]	0.2357	0	19.6	[16.9 - 22.6%]	0.765	0	6.68
Bombella_sp_ESL0385	Bombella_intestini_R52487	21.5	[18.3 - 25.1%]	0.6737	0	19.1	[16.9 - 21.5%]	0.2299	0	20.2	[17.5 - 23.3%]	0.7487	0	2.03
Bombella_sp_ESL0385	Bombella_sp_nov_TMW21880	22.4	[19.2 - 26.1%]	0.6518	0	18.9	[16.7 - 21.3%]	0.2325	0	20.9	[18.1 - 23.9%]	0.7328	0	2.42
Bombella_sp_ESL0385	Bombella_sp_nov_TMW21889	20.4	[17.2 - 24%]	0.7011	0	18.7	[16.5 - 21.1%]	0.2353	0	19.3	[16.6 - 22.4%]	0.7715	0	7.52
Bombella_sp_ESL0385	Parasaccharibacter_apium_AS1	95.3	[93.1 - 96.9%]	0.0491	98.74	47.4	[44.8 - 50%]	0.078	12.69	87.8	[84.9 - 90.3%]	0.1233	99.06	0.28
		Formula 1				Formula 2				Formula 3				G+C difference
Query genome	Reference genome	DDH	Model C.I.	Distance	Prob. DDH >= 70%	DDH	Model C.I.	Distance	Prob. DDH >= 70%	DDH	Model C.I.	Distance	Prob. DDH >= 70%	
Bombella_sp_ESL0378	Bombella_apis_30666T	20.6	[17.4 - 24.2%]	0.6961	0	18.6	[16.5 - 21%]	0.2358	0	19.5	[16.8 - 22.5%]	0.7678	0	6.71
Bombella_sp_ESL0378	Bombella_intestini_R52487	21.6	[18.4 - 25.2%]	0.6707	0	18.9	[16.7 - 21.3%]	0.2327	0	20.3	[17.5 - 23.3%]	0.7473	0	2.06
Bombella_sp_ESL0378	Bombella_sp_nov_TMW21880	22.3	[19 - 25.9%]	0.6549	0	18.9	[16.7 - 21.3%]	0.2328	0	20.8	[18 - 23.8%]	0.7353	0	2.45
Bombella_sp_ESL0378	Bombella_sp_nov_TMW21889	20.3	[17.1 - 23.9%]	0.7032	0	18.6	[16.4 - 20.9%]	0.2368	0	19.3	[16.6 - 22.3%]	0.7735	0	7.55
Bombella_sp_ESL0378	Parasaccharibacter_apium_AS1	95.8	[93.8 - 97.2%]	0.0458	98.83	47.3	[44.8 - 49.9%]	0.0781	12.57	88.3	[85.4 - 90.7%]	0.1203	99.15	0.24
		Formula 1				Formula 2				Formula 3				G+C difference
Query genome	Reference genome	DDH	Model C.I.	Distance	Prob. DDH >= 70%	DDH	Model C.I.	Distance	Prob. DDH >= 70%	DDH	Model C.I.	Distance	Prob. DDH >= 70%	
Parasaccharibacter_apium_AS1	Bombella_apis_30666T	20.8	[17.6 - 24.4%]	0.6902	0	18.5	[16.4 - 20.9%]	0.2372	0	19.6	[16.9 - 22.7%]	0.7637	0	6.96
Parasaccharibacter_apium_AS1	Bombella_intestini_R52487	21.8	[18.6 - 25.5%]	0.6657	0	18.8	[16.7 - 21.2%]	0.2336	0	20.4	[17.7 - 23.5%]	0.7438	0	2.31
Parasaccharibacter_apium_AS1	Bombella_sp_ESL0378	95.8	[93.8 - 97.2%]	0.0458	98.83	47.3	[44.8 - 49.9%]	0.0781	12.57	88.3	[85.4 - 90.7%]	0.1203	99.15	0.24
Parasaccharibacter_apium_AS1	Bombella_sp_ESL0385	95.3	[93.1 - 96.9%]	0.0491	98.74	47.4	[44.8 - 50%]	0.078	12.69	87.8	[84.9 - 90.3%]	0.1233	99.06	0.28
Parasaccharibacter_apium_AS1	Bombella_sp_nov_TMW21880	22.3	[19.1 - 26%]	0.6535	0	18.9	[16.7 - 21.3%]	0.2326	0	20.8	[18.1 - 23.9%]	0.7341	0	2.69
Parasaccharibacter_apium_AS1	Bombella_sp_nov_TMW21889	20.3	[17.1 - 23.9%]	0.7039	0	18.5	[16.3 - 20.8%]	0.238	0	19.2	[16.5 - 22.3%]	0.7744	0	7.79
		Formula 1				Formula 2				Formula 3				G+C difference
Query genome	Reference genome	DDH	Model C.I.	Distance	Prob. DDH >= 70%	DDH	Model C.I.	Distance	Prob. DDH >= 70%	DDH	Model C.I.	Distance	Prob. DDH >= 70%	
Bombella_sp_ESL0378	Bombella_sp_ESL0385	98.3	[97.1 - 99%]	0.0264	99.27	91.8	[89.6 - 93.5%]	0.0102	96.35	98.6	[97.7 - 99.1%]	0.0363	99.95	0.03
