# Supplementary material for: Comparative Genomics of Acetic Acid Bacteria within the Genus Bombella in Light of Beehive Habitat Adaptation
Source: Microorganisms. 2022 May 20;10(5):1058. doi: 10.3390/microorganisms10051058 (PMC9147383; doi:10.3390/microorganisms10051058)
Supplement: Supplementary file 1 [file microorganisms-10-01058-s001.zip › Table S3.pdf]

Table S3: Genome accessions for contigs of type 1 polyketide synthase gene clusters

	**T1PKS Contig**
***Bombella apis* MRM1^T**	JADAQV0000000004
***Bombella apis* ESL0368**	CP046394.1
***Bombella apis* ESL0380**	WNIH01000008.1
***Bombella apis* ESL0387**	WNIK01000007.1
***Bombella apis* SME1**	WHNS01000005.1
***Bombella apis* TMW 2.1882**	NWUT03
***Bombella apis* TMW 2.1884**	QORR02
***Bombella apis* TMW 2.1886**	QORS05
***Bombella apis* TMW 2.1888**	CP030979
***Bombella apis* TMW 2.1890**	NWUV03
***Bombella apis* TMW 2.1891**	QORT03
***Bombella intestini* R52487^T**	NZ_JATM01000004
***Bombella* sp. ESL0378**	NZ_WNIF01000010
***Bombella* sp. ESL0385**	NZ_WNIJ01000001
***Bombella favorum* TMW 2.1880^T**	NWUS03
***Bombella mellum* TMW 2.1889^T**	PDLY04
***Bombella apis* A29**	NZ_LMYH01000013
***Bombella apis* AM169**	NZ_CBLY010000004
***Bombella* sp. AS1**	NZ_MEJG01000002
***Bombella apis* G773c**	NZ_CP020554.1
***Bombella apis* 3A1**	NZ_MNPT01000004
***Bombella apis* M18**	NZ_MNPS01000003
***Saccharibacter floricola* DSM 15669**	NZ_KB899335.1
***Gluconacetobacter diazotrophicus* PA1 5**	CP001189.1
***Asaia bogorensis* NBRC 16594**	NZ_AP014690.1
