# Supplementary material for: Comparative Genomics of Acetic Acid Bacteria within the Genus Bombella in Light of Beehive Habitat Adaptation
Source: Microorganisms. 2022 May 20;10(5):1058. doi: 10.3390/microorganisms10051058 (PMC9147383; doi:10.3390/microorganisms10051058)
Supplement: Supplementary file 1 [file microorganisms-10-01058-s001.zip › Table S4.pdf]

Table S4: Growth inhibition zone diameter (in mm) of antibiotic disc diffusion assay.

	**Tetracycline 30**	**Doxycycline 30**	**Ampicillin 10**	**Penicillin G 5**	**Chloramphenicol 30**	**Kanamycin 30**	**Ciprofloxacin 5**	**Meropenem 10**	**Cephazolin 30**	**Norofloxacin 10**	**Trimethoprim 5**	**Tobramycin 30**
***Bombella favorum* TMW 2.1880^T**	36	31	6	X	10	13	6	22	X	X	X	11
***Bombella mellum* TMW 2.1889^T**	28	25	9	X	8	11	6	23	X	X	X	8
***Bombella apis* TMW 2.1882**	27	23	7	X	9	11	6	19	X	X	X	10
***Bombella apis* TMW 2.1884**	31	29	11	X	18	11	X	23	X	X	X	10
***Bombella apis* TMW 2.1886**	29	25	10	X	12	10	7	22	X	X	X	9
***Bombella apis* TMW 2.1888**	33	28	6	X	13	13	X	24	X	X	X	10
***Bombella apis* TMW 2.1890**	30	24	7	X	14	11	X	23	X	X	X	7
***Bombella apis* TMW 2.1891**	30	27	7	X	9	12	X	21	X	X	X	8
***Bombella apis* MRM1^T**	X	X	7	X	14	10	X	18	X	X	X	9
***Gluconobacter oxydans* DSM46615**	25	23	X	X	X	10	X	22	X	X	X	14
